# Low HDL-C Level Is Associated with the Development of Intracranial Artery Stenosis: Analysis from the Chinese IntraCranial AtheroSclerosis (CICAS) Study

**DOI:** 10.1371/journal.pone.0064395

**Published:** 2013-05-17

**Authors:** Yining Qian, Yuehua Pu, Liping Liu, David Z. Wang, Xingquan Zhao, Chunxue Wang, Yilong Wang, Gaifen Liu, Yuesong Pan, Yongjun Wang

**Affiliations:** 1 Department of Neurology, Beijing Anzhen Hospital, Capital Medical University, Beijing, China; 2 Department of Neurology, Beijing Tiantan Hospital, Capital Medical University, Beijing, China; 3 Department of INI Stroke Center & Stroke Network, OSF Healthcare System, University of Illinois College of Medicine, Peoria, Illinois, United States of America; University of Münster, Germany

## Abstract

**Background:**

Intracranial atherosclerotic stenosis (ICAS) is an important cause of ischemic stroke worldwide. The role of high-density lipoprotein cholesterol (HDL-C) or low-density lipoprotein cholesterol (LDL-C) in the development of ICAS remains to be elucidated. In the current study, we investigated the relationship of HDL-C level and the risk of developing ICAS in Chinese patients with acute ischemic stroke.

**Methods:**

From October 2007 to June 2009, a total of 1,984 consecutive ischemic stroke patients were evaluated for the presence of symptomatic ICAS by magnetic resonance angiography (MRA). Patients were classified into two groups: intracranial steno-occlusion (ICAS group, n = 888) and non-intracranial stenosis (NICAS group, n = 1096). Serum lipid profiles were analyzed and compared between the ICAS and NICAS group.

**Results:**

Significantly more patients in ICAS group had low HDL-C level (51.6%) than in the NICAS group (42.9%, P<0.001). The observed association remained significant after adjustment for conventional risk factors [(adjusted OR 1.36; 95%CI (1.13–1.63)]. Such predictive value of low level HDL-C persisted even when LDL-C was at very low level(<1.8 mmol/L). Patients in the lowest serum HDL-C quartile (<0.96 mmol/L) had the highest risk of developing ICAS [adjusted OR 1.52; 95%CI (1.17–1.98)] compared to patients in the highest serum HDL-C quartile (≥1.32 mmol/L) after adjustments for the covariates.

**Conclusions:**

Low HDL-C level is strongly associated with the development of ICAS. There was an inverse relationship between the level of HDL-C and the risk of developing ICAS.

## Introduction

Intracranial atherosclerotic stenosis (ICAS) is an important cause of ischemic stroke worldwide [1]. ICAS is responsible for 8% to 10% of all ischemic strokes in the United States [2], but accounts for 33% to 54% of all ischemic strokes in Asia [3]. In China, ICAS may be the cause of 37% to 51% of all strokes or transient ischemic attacks (TIA) [4], [5]. Multiple modifiable risk factors, such as smoking, hypertension, diabetes mellitus (DM), and metabolic syndrome, may all contribute to the development of ICAS [6], [7]. However, the relationship between dyslipidemia and ICAS remains to be elucidated [8]. In previous reports, high serum lipoprotein (a) has been associated with the development of ICAS [9]. In China, low HDL-C is one of the most common types of dyslipidemia [10]. The role of HDL-C in ICAS has not been fully studied. Since low HDL-C level is strongly associated with cardiovascular events [11], we hypothesized that low HDL-C may be related to a high incidence of ICAS in the Chinese population. To test this hypothesis, we investigated the lipid profiles and MRA in order to evaluate Chinese acute ischemic stroke patients with ICAS. In addition, patients with low serum HDL-C levels,with or without ICAS, were compared to those in the Chinese Intra-Cranial AtheroSclerosis study (CICAS).

## Methods

### Ethics Statement

This protocol was approved by the ethics committee of the Beijing Tiantan Hospital of Capital Medical University and was performed in accordance with the guidelines of the Helsinki Declaration. After ethical approval of Tiantan Hospital was obtained and distributed to each center, the ethical approval took effect automatically in each center. All patients or their legal representatives provide their written informed consent form (ICF).

### Patients

CICAS is a prospective multicenter hospital based cohort study to investigate the distribution of intracranial atherosclerosis by using MRA findings in Chinese patients with acute cerebral ischemia. From October 2007 to June 2009, consecutive patients from 22 hospitals were recruited according to the following criteria: 18 to 80 years old who had an acute ischemic stroke within seven days of symptom onset. Exclusion criteria included: presumed cardioembolic stroke, unfit for MRA study, unstable medical conditions, or disabled (modified Rankin scale>2) prior to admission. Acute ischemic stroke was diagnosed according to the World Health Organization criteria combined with magnetic resonance imaging (MRI) findings.

On admission, baseline data, including age, gender, medical history and physical examination were collected. All patients had detailed clinical evaluation,neurological examination, relevant laboratory tests, cardiac evaluation, MRI, three-dimensional time of flight magnetic resonance angiography (3D TOF MRA) of the intracranial circulation. Extracranial carotid vessels were examined by duplex color Doppler ultrasound. Cardiac evaluation included 24-hour electrocardiogram, transthoracic and transesophageal echocardiography. Transesophageal echocardiography was performed on the same day in cases where a high-risk cardiac source of embolism was clinically suspected.

From October 2007 to June 2009, 3,580 patients were registered. In order to exclude any potential confounding factors that may interfere with the final analysis, only patients with ICAS were included. We excluded 325 patients who had cardioembolism and 391 patients with incomplete cerebrovascular diagnostic workup. In addition the following patients were excluded: 41 patients with serious debilitating terminal illnesses, 71 patients with previous use of lipid-lowering drugs, 79 patients without serum lipid levels, and 287 TIAs. To minimize confounding factors, 141 patients with only extracranial carotid stenosis and 230 patients with both intra and extracranial carotid stenosis were also excluded. The remaining 1,984 patients (1,323 men and 661 women) with acute ischemic strokes were entered into the final analysis.

Clinical information from each patient included age, gender, hypertension, diabetes mellitus (DM), and hyperlipidemia. National Cholesterol Education Program (NCEP) Expert Panel on Detection, Evaluation, and Treatment of High Blood Cholesterol in Adults (Adult Treatment Panel III) guidelines were used to identify people with low values of HDL-C (<1.03 mmol/L in men, <1.30 mmol/L in women), high values of total cholesterol (≥5.18 mmol/L),high values of triglycerides (TG, ≥1.7 mmol/L), nonoptimal LDL-C values (≥2.59 mmol/L), and Non-HDL-C level (≥3.36 mmol/L) [12]. A history of cerebral ischemia (including a history of ischemic stroke and TIA), heart disease, smoking and peripheral arterial disease was also collected.

### MRI and MRA

All patients underwent conventional MRI and 3D TOF MRA on a 1.5 or 3.0 T magnetic resonance scanner within 3 days following admission. The acquisition parameters of MRI consisted of transverse T2/T1-weighted, fluid-attenuated inversion recovery sequences and sagittal T1 with 5 mm thickness slices without interslice gaps. Diffusion weighted imaging (DWI) was obtained in the transverse plane using a single-shot echo planar, spinecho pulse sequence. 3D TOF MRA was performed using a repetition time (TR) of 25 milliseconds (msec) and an echo time (TE) of 2 msec. All measurements were made with Wiha DigiMax Digital Calipers 6′ (Germany) at a resolution of 0.01–0.03 mm for 0–100 mm. The degree of stenosis was calculated by the published method in the Warfarin–Aspirin Symptomatic Intracranial Disease Study [13]. We assessed the following arterial segments: bilateral intracranial internal carotid artery (ICA), anterior cerebral artery (ACA) A1/A2, middle cerebral artery (MCA) M1/M2, posterior cerebral artery (PCA) P1/P2 and basilar artery (BA). For the internal carotid artery, an intracranial location was defined when the stenotic lesion was distal to the ophthalmic artery. According to the severity of stenosis, all participants were categorized into two subtypes based on the angiographic findings: (1) No intracranial stenosis (NICAS group, those with 0–49% diameter stenosis in the intracranial arteries); (2) Intracranial steno-occlusion (ICAS group, those with 50–100% diameter steno-occlusion in the intracranial arteries [14]. The ICAS group was further categorized into the mild-stenosis group (50–69%) and a severe steno-occlusion group (70–100%), and single vessel or multi-vessel stenosis group.

All MRI/MRA images were stored in digital format and read independently by two radiologists (XY Zou and Y Soo) who were blinded to the results of the clinical information. The level of agreement for the presence of stenosis on angiography was acceptable (kappa = 0.91, p<0.05). A third reader's opinion was obtained when there was disagreement between the two primary readers.

### Biochemical analysis

Lipid profiles were obtained after 12-hours fasting following admission. Serum TC and TG concentrations were assayed by routine enzymatic methods. The concentration of LDL-C and HDL-C were determined by a direct homogeneous assay. All 22 cooperative hospitals utilized the same kind of laboratory test to check the lipid profiles. All labs conformed to the quality standard set by the National Center for Clinical Laboratory of China.

### Statistical analysis

All statistical tests were performed with SAS software version 9.1 (SAS Institute Inc, Cary, NC). Independent-samples t test or Wilcoxon test were used for comparison of continuous variables, and χ2 test or Fisher's exact test were used for comparison of categorical variables. Odds ratios (OR) and their 95% confidence intervals (CI) for the presence of ICAS associated with various dyslipidemia subgroups were calculated using logistic regression analysis adjusted on gender, age, hypertension, DM and history of stroke or transient ischemic attack. Ordinal logistic regression analysis was performed to examine the relationship between serum HDL-C level and the severity of ICAS. Multivariate OR with 95% CI for the relationship between serum HDL-C levels and ICAS were calculated with binary logistic regression. The highest (fourth) quartile was used as a reference. A Cochran-Armitage trend test was performed to examine the relationship between serum HDL-C level and ICAS. In all comparisons, P<0.05 represented a statistically significant difference.

## Results

**Figure 1:**
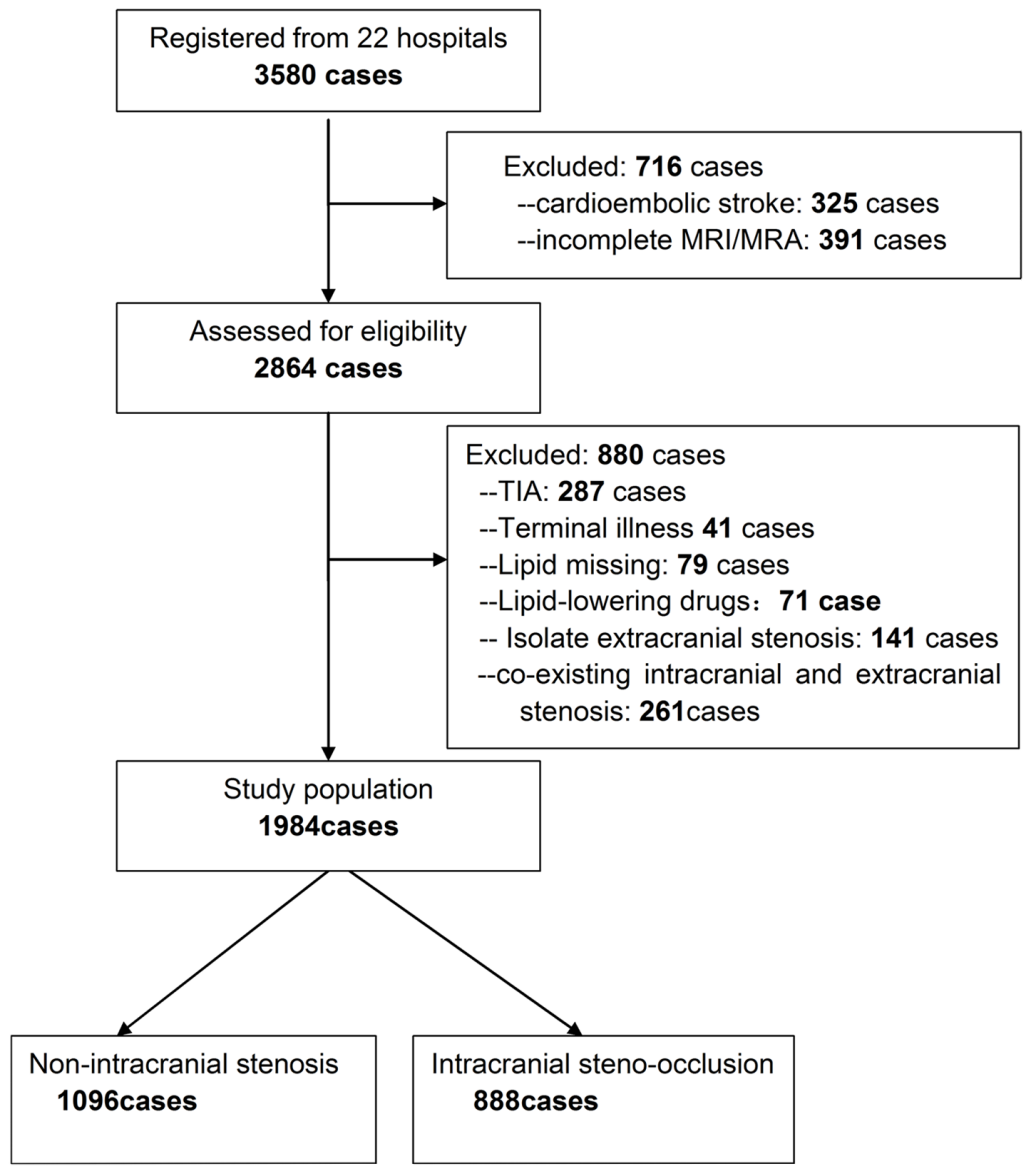
Flow diagram of enrollment.

There were no missing data in all 1,984 patients enrolled. Among them, 888 (45%) were classified into the symptomatic ICAS group (with 50–100% steno-occlusion) and 1,096 (55%) into the NICAS group (Figure 1). Baseline characteristics are shown in Table 1 and Table 2. There were 1,323 men and 661 women in the present study, and the average age was 61.6±11.3 years. Compared with the NICAS group, the ICAS group had significantly higher rate of hypertension (81.2% vs. 77.5%, p = 0.04), DM (38.7% vs. 30.0%, p<0.001), stroke or TIA history (26.0% vs. 22.0%, p = 0.04), and lower level of HDL-C [(1.14±0.31) mmol/L vs. (1.18±0.33) mmol/L, p = 0.007]. Older age (>65 years) was a risk factor for ICAS (42.9% vs. 38.4%, p = 0.04).

**Table 1:** Baseline clinical characteristics of the 1984 patients.

Characteristic†, num(%)	Total n = 1984(100)	Non-intracranial stenosis n = 1096(55)	Intracranial steno-ocllusion n = 888(45)	P -value
Gender, male	1323(66.7)	751(68.5)	572(64.4)	0.05
Age, mean(SD) (year)	61.6±11.3	61.0±11.3	62.2±11.3	0.02
>65years	802(40.4)	421(38.4)	381(42.9)	0.04
Hypertension	1570(79.1)	849(77.5)	721(81.2)	0.04
Diabetes	673(33.9)	329(30.0)	344(38.7)	<0.001
Dislipidemia	1639(82.6)	906(82.7)	733(82.6)	0.94
Heart disease	140(7.1)	76(6.9)	64(7.2)	0.81
Peripheralarterial disease	14(0.7)	8(0.7)	6(0.7)	0.89
Stroke or TIA history	472(23.8)	241(22.0)	231(26.0)	0.04
Smoking	730(36.8)	414(37.8)	316(35.6)	0.31
Heavy drink	97(4.9)	56(5.1)	41(4.6)	0.61
BMI≥25 kg/cm^2^	619(40.5)	348(41.2)	271(39.6)	0.54
BMI, mean(SD), kg/cm^2^	24.4±3.1	24.5±3.2	24.5±3.1	0.76
Systolic blood pressure mean(SD), mmHg	154.9±23.6	154.6±23.4	155.4±23.9	0.45
Diastolic blood pressure mean(SD), mmHg	89.8±13.6	89.9±13.4	89.8±13.9	0.78

TIA, transient ischemic attacks; BMI, body mass index; SD, Standard Deviation.

† Data are given as number (percentage) except where otherwise indicated.

**Table 2:** Baseline lipid characteristics of the 1984 patients.

Characteristic†	Total n = 1984(100)	Non-intracranial stenosis n = 1096(55)	Intracranial steno-ocllusion n = 888 (45)	P Value
TC, mean(SD),mmol/L	4.74±1.17	4.75±1.12	4.71±1.24	0.45
TC≥5.18 mmol/L	626(31.6)	356(32.5)	270(30.4)	0.32
TG, mean(IQR),mmol/L	1.10(1.5,2.11)	1.10(1.48,2.12)	1.11(1.51,2.10)	0.99
TG≥1.7 mmol/L	784(39.5)	436(39.8)	348(39.2)	0.79
LDL-C, mean(SD),mmol/L	2.93±0.95	2.92±0.94	2.94±0.97	0.69
LDL-C≥2.59 mmol/L	1258(63.4)	693(63.2)	565(63.6)	0.86
HDL-C, mean(SD),mmol/L	1.16±0.32	1.18±0.33	1.14±0.31	0.007
‡HDL-C<1.03 mmol/L	928(46.8)	470(42.9)	458(51.6)	0.0001
Non-HDL-C mean(SD),mmol/L	3.57±1.13	3.57±1.08	3.57±1.19	0.98
Non-HDL-C≥3.36 mmol/L	1136(57.2)	628(57.3)	508(57.2)	0.97

SD, Standard Deviation; IQR, inter-quartile range; Non-HDL-C: non high-density lipoprotein cholesterol.

† Data are given as number (percentage) except where otherwise indicated.

‡ HDL-C<1.03 mmol/L for men, <1.30 mmol/L for women.

Among the ICAS group, 226 (25.4%) patients had mild stenosis (50–69%), 186 (20.9%) had severe stenosis (70–99%), and 476 (53.6%) had occlusion, 544 (61.3%) had single vessel ICAS, and 344 (38.7%) had multi-vessel stenosis. Complete occlusion was found in 83 (60%) ACA, 322 (56%) MCA, 183 (51%) PCA, 65 (60%) BA and 56 (80%) intracranial ICA. Severe stenosis (70–99%) was found in 34 (25%) ACA, 122 (21%) MCA, 83 (23%) PCA, 21(19%) BA, and 9 (13%) intracranial ICA. Mild stenosis (50–69%) was found in 20 (15%) ACA, 128(22%) MCA, 90 (25%) PCA, 22 (20%) BA, and 5 (7%) intracranial ICA (Table 3).

**Table 3:** Distribution of intracranial atherosclerotic stenosis.

Distribution of ICAS	50–70% stenosis	70–99% stenosis	Occlusion
ACA (n = 137)	20(15)	34(25)	83(60)
MCA (n = 572)	128(22)	122(21)	322(56)
PCA (n = 356)	90(25)	83(23)	183(51)
Ba (n = 108)	22(20)	21(19)	65(60)
intracranial ICA (n = 70)	5(7)	9(13)	56(80)

ICAS, intracranial atherosclerotic stenosis; ACA, anterior cerebral artery; MCA, middle cerebral artery; PCA, posterior cerebral artery; BA, basilar artery; ICA, internal carotid artery.

Univariate analysis showed that low HDL-C level was closely associated with the development of ICAS (p<0.0001). In the ICAS group, more patients had low HDL-C levels (51.6%, n = 458) than in the NICAS group (42.9%, n = 470, P<0.0001, Table 2).

**Figure 2:**
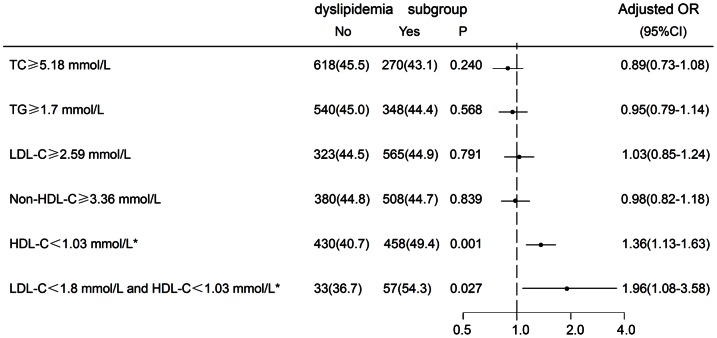
Adjusted odds ratios and 95% confidence intervals for ICAS associated with different dyslipidemia subgroups. Odds ratios for ICAS adjusted for gender, age, hypertension, diabetes, and history of stroke or transient ischemic attack. OR(Odds Ratios); 95%CI(Confidence Intervals); ICAS, intracranial atherosclerotic stenosis; TC, Total Cholesterol; LDL-C, low density lipoprotein cholesterol; TG, triglyceride; HDL-C, high-density lipoprotein cholesterol; Non-HDL-C: non high-density lipoprotein cholesterol; ^*^ HDL-C<1.03 mmol/L for men, <1.30 mmol/L for women, P<0.001.

In the multivariate logistic regression model, after adjusting for gender, age, hypertension, DM, history of stroke or TIA, low HDL-C level remained an independent risk factor for the development of ICAS (adjusted OR, 1.36; 95% CI 1.13–1.63). In contrast, other forms of dyslipidemia, such as high TG level (≥1.7 mmol/L), high TC level (≥5.18 mmol/L), LDL-C level (≥2.59 mmol/L), and Non-HDL-C level (≥3.36 mmol/L) had no significant association with ICAS. The relationship between low HDL-C level and ICAS maintained significant even in patients whose LDL-C level was less than 1.8 mmol/L [adjust OR 1.96; 95%CI (1.08–3.58)] (Figure 2).

About 112 (49.6%) patients with low HDL-C level were in the mild (50–69%) ICAS group (n = 226), and 346 (52.3%) patients with low HDL-C level were in the severe steno-occlusion (70–100%) ICAS group (n = 662), suggesting an inverse relationship between HDL-C level and the severity of stenosis. Ordinal logistic regression showed a significant inverse association of the levels of HDL-C and the severity of ICAS [adjusted OR 0.68; 95% CI (0.51–0.89); p = 0.0064].

Since the presence of multiple intracranial stenosis may be an indication of the severity of ICAS, the relationship of levels of HDL-C in patients with multiple intracranial stenosis was analyzed. About 261 (48.0%) patients with low HDL-C level were in the single vessel ICAS group (n = 544), and 197 (57.3%) of patients with low HDL-C level were in the multi-vessel ICAS group (n = 344), suggesting a strong correlation of low serum HDL-C level and the development of multiple ICAS. Ordinal logistic regression analysis also showed a significant inverse association of the levels of HDL-C and the multi-vessel of ICAS [adjusted OR 0.65; 95%CI (0.49–0.86); P = 0.0025].

As shown in Table 4, serum HDL-C levels were stratified into quartiles, with the highest quartile serving as the reference. The risk of developing ICAS was increased across the quartiles. Compared with the highest quartile and after adjustments for gender, age, hypertension, DM, stroke or TIA history (P = 0.01), the odds of developing ICAS was 52% higher in the lowest quartile [(adjusted OR 1.52; 95% CI (1.17–1.98)]. A Cochran-Armitage trend test showed a strong correlation between low serum HDL-C level and high risk of developing ICAS (Z = −3.8825, P = 0.0001). Use the untransformed metric variable of HDL-C in binary logistic regression,the odds ratios of developing ICAS associated with each mmol/L increase of HDL-C was 0.66, 95%CI(0.49–0.89), P = 0.0056.

**Table 4:** Odds ratios (OR) and their 95% confidence intervals (CI) for the binary logistic regression analyses on association between serum HDL-C levels and ICAS.

HDL-C† Quartile (mmol/L)	Number of ICAS	Incidence of ICAS(%)	Univariate OR (95%CI)	Multivariatre OR (95%CI)	P-value
4^th^(≥1.32)	201	39.72	-	-	
3^rd^(1.12–1.32)	211	42.20	1.11	1.12(0.87–1.45)	0.15
2^nd^(0.96–1.12)	240	48.19	1.41	1.46(1.13–1.89)	0.05
1^st^(<0.96)	236	49.16	1.47	1.52(1.17–1.98)	0.01*

Odds ratios for ICAS adjusted for gender, age, hypertention, diabetes, and history of stroke or transient ischemic attack. ICAS, intracranial atherosclerotic stenosis.

† HDL-C<1.03 mmol/L for men, <1.30 mmol/L for women.

* P<0.01.

## Discussion

In our study, we have found that the incidence of ICAS in acute ischemic strokes was 45% in the studied population. This finding was consistent with previous reports of high prevalence of ICAS in the Chinese population [15]. However, the most significant finding of our study was that patients with ICAS more likely had low HDL-C levels (51.6%) compared to those in the NICAS group (42.9%) (P<0.001).

This is the first study to examine specifically the relationship between HDL-C level and risk of developing ICAS in patients with acute ischemic stroke. Despite several previous studies indicating an independent inverse relationship between serum HDL-C levels and overall stroke risk [16], [17], no study has demonstrated the specific relationship between low HDL-C level and the risk of ICAS. The results of our study suggested that low HDL-C level, not high LDL-C level, was independently associated with an increased risk of developing ICAS. Furthermore, there was a quantitative interaction. In our study, we have found that the lower the level of HDL-C, the higher the chance of developing ICAS. As shown in Table 4, patients with the lowest quartile of HDL-C had a 52% increased risk of ICAS as compared to the highest quartile, after adjusting for the covariates. Our data also implied an inverse relationship between HDL-C level and the severity of stenosis. Low HDL-C level highly correlated with severity of stenosis of occlusion, indicating a protective role of HDL-C against ICAS. In contrary, we found no relationship between high LDL-C level and the risk of ICAS, similar to a recent report in the Korean population [18].

Our study also showed that low HDL-C level remains as an independent predictor of ICAS even in patients with very low levels of LDL-C. One meta analysis showed that increasing the concentration of HDL-C with statin therapy can slow and even reverse the progression of coronary atherosclerosis [19]. The findings of SAMMPRIS have suggested that aggressive medical management should be the first-choice for patients with symptomatic high-grade ICAS [20]. These reports remind us that raising HDL-C level perhaps should be one of the main therapeutic strategies for the prevention of ICAS in addition to controlling LDL-C level.

The exact underlying mechanism of how low level HDL-C may cause ICAS is unclear. However, it is well established that HDL–C may protect against atherosclerosis by promoting cholesterol efflux from macrophages in the artery wall. In addition, HDL-C may also reduce oxidation, vascular inflammation and thrombosis, improve endothelial function, promote endothelial repair, enhance insulin sensitivity and promote insulin secretion by pancreatic beta islet cells [21]. One study postulated that intracranial arteries have greater antioxidant enzyme activity compared to the extracranial arteries [22]. In our study, patients in the ICAS group had a higher incidence of low HDL-C, indicating low antioxidant activity [23] as compared to those in the NICAS group (51.6% vs. 42.9%). However, no such differences were observed in the patients with high LDL-C levels. Therefore, a more selective loss of antioxidant activity in ICAS may explain why low HDL-C, not high LDL-C, increases the risk of developing ICAS.

Several epidemiologic studies, such as the Framingham Heart Study, US Physicians' Health Study, Prospective Cardiovascular Münster (PROCAM) Study, and Atherosclerosis Risk in Communities (ARIC) Study, have demonstrated an inverse relationship between HDL-C level and cardiovascular risk [24]–[27]. In contrast, several clinical trials that evaluated HDL-C raising strategies, such as AIM-HIGH trial (niacin), ACCORD trial (fenofibrate), and ILLUMINATE trial (torcetrapib), have failed to demonstrate any clinical benefit [28]–[30]. These disappointing results remind us that perhaps the strategy of raising HDL-C serum levels alone is insufficient as a therapeutic target. It has become clear that the biological functions of HDL are altered in patients with coronary disease or diabetes. It may as well be altered in ICAS. Such change may have rendered HDL dysfunctional or even proinflammatory and thus promote atherosclerosis [31], [32]. Therefore, for HDL-C to be protective against ICAS, both the quality and quantity of HDL are important. A large prospective trial to prove this finding and concept is currently being designed in China.

Our study has several limitations. First, all participating hospitals are tertiary hospitals, which potentially may have a higher percentage of ICAS patients. Second, serum apolipoproteins (like ApoA1) and fractions of HDL-C were not obtained. Third, MRA may have a tendency to overestimate the degree of intracranial stenosis. Lastly, MRA could not evaluate smaller cerebral arteries.

## Conclusions

Our study provides convincing data that low HDL-C level is associated with the development of ICAS in Chinese patients with acute ischemic stroke. There was a strong inverse relationship between the level of HDL-C and the risk of developing ICAS. Further prospective randomized controlled trials are needed to confirm our observations.
